# Association of tryptophan pathway metabolites with mortality and effectiveness of nutritional support among patients at nutritional risk: secondary analysis of a randomized clinical trial

**DOI:** 10.3389/fnut.2024.1335242

**Published:** 2024-02-15

**Authors:** Jacqueline Ritz, Carla Wunderle, Franziska Stumpf, Rahel Laager, Pascal Tribolet, Peter Neyer, Luca Bernasconi, Zeno Stanga, Beat Mueller, Philipp Schuetz

**Affiliations:** ^1^Medical University Department, Division of General Internal and Emergency Medicine, Cantonal Hospital Aarau, Aarau, Switzerland; ^2^Medical Faculty of the University of Basel, Basel, Switzerland; ^3^Department of Health Professions, Bern University of Applied Sciences, Bern, Switzerland; ^4^Department of Nutritional Sciences and Research Platform Active Aging, University of Vienna, Vienna, Austria; ^5^Institute of Laboratory Medicine, Cantonal Hospital Aarau, Aarau, Switzerland; ^6^Division of Diabetes, Endocrinology, Nutritional Medicine, and Metabolism, Bern University Hospital and University of Bern, Bern, Switzerland

**Keywords:** tryptophan, kynurenine, serotonin, biomarker, metabolomics, malnutrition, nutritional support

## Abstract

Tryptophan is an essential amino acid and is the precursor of many important metabolites and neurotransmitters. In malnutrition, the availability of tryptophan is reduced, potentially putting patients at increased risks. Herein, we investigated the prognostic implications of the tryptophan metabolism in a secondary analysis of the Effect of Early Nutritional Support on Frailty, Functional Outcomes, and Recovery of Malnourished Medical Inpatients Trial (EFFORT), a randomized, controlled trial comparing individualized nutritional support to usual care in patients at risk for malnutrition. Among 238 patients with available measurements, low plasma levels of metabolites were independently associated with 30-day mortality with adjusted hazard ratios (HR) of 1.77 [95% CI 1.05–2.99, *p* 0.034] for tryptophan, 3.49 [95% CI 1.81–6.74, *p* < 0.001] for kynurenine and 2.51 [95% CI 1.37–4.63, *p* 0.003] for serotonin. Nutritional support had more beneficial effects on mortality in patients with high tryptophan compared to patients with low tryptophan levels (adjusted HR 0.61 [95% CI 0.29–1.29] vs. HR 1.72 [95% CI 0.79–3.70], *p* for interaction 0.047). These results suggest that sufficient circulating levels of tryptophan might be a metabolic prerequisite for the beneficial effect of nutritional interventions in this highly vulnerable patient population.

## 1 Introduction

Malnutrition is frequent in medical inpatients with a prevalence of more than 30% and is strongly associated with an increased risk for mortality, morbidity, functional decline, and impairments in quality of life (1–3). The Effect of Early Nutritional Support on Frailty, Functional Outcomes, and Recovery of Malnourished Medical Inpatients Trial (EFFORT) and other trials demonstrated that early individualized nutritional support improves clinical outcomes in patients at risk for malnutrition (4–6). Still, recent data suggest that individual response to nutrition treatment may vary and that not all patients show clinical benefit when nutritional support is initiated. In fact, several novel analyses have found disease-related conditions and blood biomarkers that identified patients with a strong benefit from nutritional support (7–9). For example, patients with high levels of inflammation have shown to benefit less from nutritional support compared to patients with moderate or low inflammation levels (8). These findings are consistent with previous trials conducted in critically ill patients (10, 11). To further personalize and improve the efficiency of nutritional support, metabolites from different metabolic pathways, including amino acids, may also serve as biomarkers to predict treatment response.

Herein, tryptophan, an essential amino acid whose dietary intake is necessary for protein synthesis, and its metabolites are potential nutritional biomarkers. Tryptophan is the precursor of neurotransmitters, such as serotonin and melatonin, and other physiologically important metabolites involved in redox reactions or the citrate cycle (12). Deficient protein intake results in low tryptophan plasma levels and thus low tryptophan availability (13, 14). Furthermore, tryptophan level is known to decrease with age (15). About 95% of tryptophan is metabolized to kynurenine by the rate-limiting enzymes tryptophan-2,3-dioxygenase (TDO) and indoleamine-2,3-dioxygenase (IDO) (16, 17). IDO is localized in extrahepatic tissue and is induced, e.g., by interferon-gamma. Initially, this was thought to be a defense mechanism for reducing the availability of tryptophan to intracellular parasites, cancer cells, or pathogens (18, 19). However, recent studies have demonstrated that IDO activity inhibits T cell proliferation and like this modulates inflammatory response (20). Tumor cells take advantage of this mechanism by expressing IDO and thus suppress antitumor immunity (21). Activation of the tryptophan/kynurenine pathway with high IDO activity has been observed in several clinical conditions, such as infection, inflammation, and malignant disease and may contribute to disease severity and adverse clinical outcomes (22–24). Less than 5% of tryptophan is metabolized to serotonin and other neurotransmitters. Tryptophan hydroxylase (TPH) catalyzes the oxygenation of tryptophan to 5-hydroxytryptophan (5-HTP) which, in a second step, is decarboxylated to serotonin. In addition to central effects as a neurotransmitter, serotonin leads to vasoconstriction and endothelial hyperpermeability in the periphery (25). Accordingly, activation of the serotonin pathway was found in several diseases including chronic obstructive pulmonary disease (COPD), coronary artery disease and sepsis and each time associated with severity and outcomes (22, 26, 27). Yet, today, little is known about the role of tryptophan and its metabolites in malnutrition. Herein, we investigated the association of tryptophan metabolites with clinical outcomes and the response to nutritional therapy among patients at risk for malnutrition.

## 2 Methods

### 2.1 Study design

This is a secondary analysis of EFFORT, a multicenter, pragmatic, randomized, controlled clinical trial comparing individualized nutritional support to usual care in patients at risk for malnutrition (6). The study was conducted in eight Swiss hospitals between April 2014 and February 2018. The Ethics Committee of Northwest and Central Switzerland (EKNZ) approved the study protocol in January 2014 (registration ID 2014_001). The trial was registered at ClinicalTrial.gov (NCT02517476) in August 2015.

### 2.2 Patient population

The EFFORT trial enrolled 2,088 patients. Inclusion criteria were: age ≥ 18 years, Nutritional Risk Screening [NRS 2002; (28)] total score of ≥3 points and an expected hospital stay of more than 4 days. The NRS 2002 includes assessment of the patient’s nutritional status (based on weight loss, body mass index, and food intake), disease severity, and age. All patients or their authorized representatives provided written informed consent. Patients initially admitted to intensive care or surgical units were excluded. Other exclusion criteria were inability of oral food intake, nutritional support on admission, anorexia nervosa, acute pancreatitis, acute liver failure, cystic fibrosis, terminal condition, stem-cell transplantation, and after gastric bypass surgery.

In this secondary analysis, we included patients from the Medical University Clinic at the Cantonal Hospital Aarau with available measurements of tryptophan, serotonin, and kynurenine.

### 2.3 Study intervention

Patients were randomly assigned (1:1) to receive either individualized nutritional support (intervention group) or usual hospital food (control group). In the intervention group, nutritional support was initiated as soon as possible after randomization but within 48 h after hospital admission. Individualized nutritional goals were defined for each patient upon hospital admission by a trained registered dietitian based on a previous consensus protocol following international guidelines, detailed elsewhere (29, 30). The intervention started with oral nutritional support. However, if less than 75% of caloric and protein targets were reached within 5 days; nutritional support was escalated to enteral or parenteral feeding. Every 24–48 h, the nutritional intake was re-assessed. Patients in the control group received standard hospital food without nutritional consultation or recommendation for additional nutritional support.

### 2.4 Analysis of blood biomarkers

Blood samples were systematically collected upon study inclusion for later measurement of biomarkers by drawing a venous blood sample into BD Vacutainer Serum Separator Tubes. Samples were immediately processed, i.e., sent to the laboratory and centrifugated, frozen in aliquots, and stored under temperature control at −80°C until further analysis. Admission plasma metabolites were analyzed from February to April 2019 by liquid chromatography coupled to tandem mass spectrometry (LC-MS/MS). An Ultimate 3000 HPLC (Thermo Fisher, San Jose, United States) system coupled to a Sciex 5500 quadrupole linear ion trap mass spectrometer (Sciex, Darmstadt, Germany) and the AbsoluteIDQ® p180 kit (BIOCRATES Life Sciences AG, Innsbruck, Austria) were used (31–33). An inter-laboratory assessment of the commercially available kit for targeted metabolomics showed reliability of the metabolomics assay (34). Measurement variation was monitored via three different quality control levels as provided by the kit manufacturer (Biocrates, Innsbruck, Austria) which were analyzed in the same way as the samples. Variation was within of the manufacturer’s specifications and no relevant batch effects were observed with the final measurements. One questionable plate was rerun after a technical issue with the chromatographic instrument had been identified.

The ratio of kynurenine to tryptophan was used as a sensitive indicator of IDO activity (22, 35). The same principle was applied to calculate activity of TPH (ratio of serotonin to tryptophan).

### 2.5 Endpoints

The primary outcome was 30-day all-cause mortality. Secondary endpoints were all-cause mortality within 180 days and adverse clinical outcomes within 30 days including all-cause mortality, admission to the intensive care unit, hospital readmission after discharge, major complications (including nosocomial infection, respiratory failure, major cardiovascular event, acute renal failure, or gastro-intestinal failure), and a decline in functional status of ≥10% measured by the Barthel index. The scores of Barthel index range from 0 to 100 points. Higher scores indicate better performance of daily activities. Follow-up interviews at day 30 and 180 were conducted with blinding to group assignment. Mortality during follow-up was verified by relatives or the patient’s family doctor.

### 2.6 Statistical analysis

All statistical analyses were performed using Stata 17. A *p* value of <0.05 was considered to indicate statistical significance. Due to the limited sample size, we did not adjust for multiple testing. Optimal cut off values for each metabolite were calculated using ROC analysis according to the Liu method (36) for 30-day mortality because no reference values in this patient population was available.

Continuous variables were expressed as mean values with the corresponding standard deviation (SD) and categorical variables as percentages or numbers. We compared frequencies using Pearson’s chi-squared test and continuous variables using a two-sample *t*-test. We used logistic regression models for binary outcomes reporting odds ratios (OR) and linear regression models for continuous outcomes reporting coefficients (Coef) with the corresponding 95% confidence interval (CI). For time-to-event data we used cox regression models reporting hazard ratios (HR). We adjusted for comorbidities, age, sex, randomization group, and NRS 2002 total score. Age adjusted Charlson Comorbidity Index (CCI) (37) was used to quantify comorbidities and age for each patient. Kaplan Meier curves were used to graphically represent the survival rate.

## 3 Results

### 3.1 Patient characteristics

**Figure 1:**
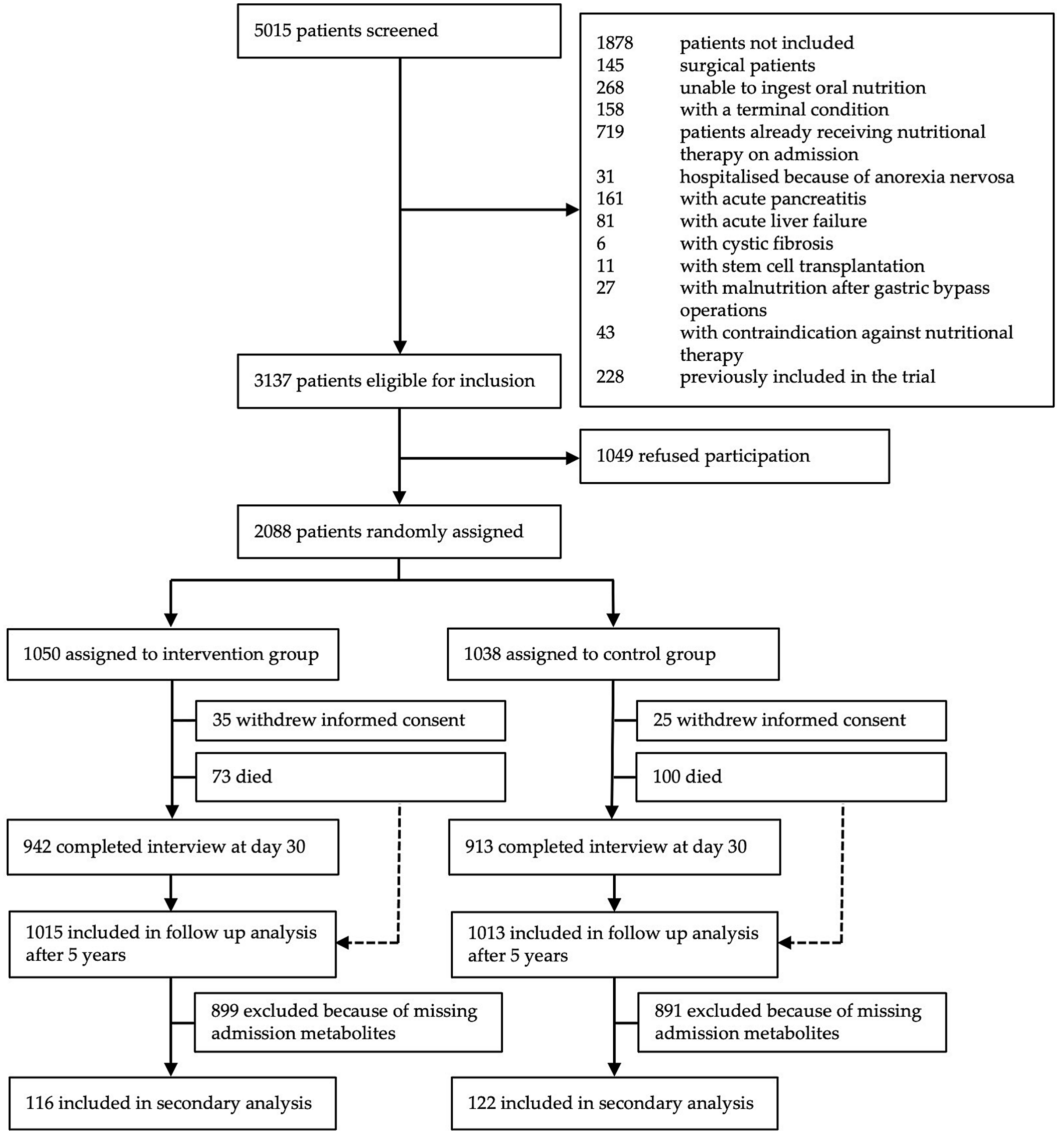
Study flow chart.

From the initial cohort including 2,088 patients, metabolite measurements were performed in samples of 238 patients from one study center participating in this sub-study. Of these 238 patients, 116 were assigned to the intervention group and 122 to the control group (Figure 1).

In total, we had 238 tryptophan, 236 kynurenine, and 213 serotonin measurements in our cohort. Calculated cut off values based on the ROC curve analysis were 36.2 μmol/L for tryptophan, 3.4 μmol/L for kynurenine, 0.25 μmol/L for serotonin, 66.6 for IDO, and 4.3 for TPH. The mean value of tryptophan was 41.1 μmol/L (SD 16.5) and 93 patients (39.1%) had tryptophan levels below the calculated cut off value (36.2 μmol/L). The mean patient age was 73 years and 57.6% of patients were male. Per inclusion criteria, all patients were at risk for malnutrition with NRS 2002 total scores of 3 (*N* = 62, 26.1%), 4 (*N* = 80, 33.6%), or above 5 (*N* = 96, 40.3%). Most patients were polymorbid and the most common diagnoses at admission were cancer (*N* = 75, 31.5%), infection (*N* = 65, 27.3%), and cardiovascular disease (*N* = 24, 10.1%). The 30-day all-cause mortality was 24.4% (*N* = 58). Additional baseline characteristics of the overall population and stratified according to tryptophan levels are shown in Table 1.

**Table 1:** Baseline characteristics.

	Overall	High tryptophan levels (≥36.2 μmol/L)	Low tryptophan levels (<36.2 μmol/L)	*p* value
Sociodemographic				
Patient number, *n* (%)	238	145 (60.9%)	93 (39.1%)	
Male sex, *n* (%)	137 (57.6%)	87 (60.0%)	50 (53.8%)	0.34
Age, mean (SD)	73.4 (13.5)	73.8 (13.1)	72.7 (14.3)	0.57
Nutritional assessment, mean (SD)				
Body mass index (kg/m^2^)	24.3 (5.0)	24.6 (5.1)	23.7 (4.7)	0.18
Mean body weight (kg)	68.7 (14.9)	70.2 (15.3)	66.3 (14.0)	**0.05**
Height (cm)	168.1 (8.6)	168.9 (8.6)	166.9 (8.5)	0.078
NRS 2002 total score, *n* (%)				0.78
3 points	62 (26.1%)	40 (27.6%)	22 (23.7%)	
4 points	80 (33.6%)	47 (32.4%)	33 (35.5%)	
≥5 points	96 (40.3%)	58 (40.0%)	38 (40.9%)	
Musclefunction, mean (SD)				
Handgrip strength (kg)	23.7 (11.6)	24.3 (11.5)	22.9 (11.6)	0.47
Admission diagnosis, *n* (%)				
Infection	65 (27.3%)	33 (22.8%)	32 (34.4%)	**0.049**
Cancer	75 (31.5%)	39 (26.9%)	36 (38.7%)	0.056
Cardiovascular disease	24 (10.1%)	18 (12.4%)	6 (6.5%)	0.14
Frailty	13 (5.5%)	8 (5.5%)	5 (5.4%)	0.96
Lung disease	11 (4.6%)	11 (7.6%)	0 (0.0%)	**0.007**
Gastrointestinal disease	13 (5.5%)	10 (6.9%)	3 (3.2%)	0.22
Neurological disease	4 (1.7%)	3 (2.1%)	1 (1.1%)	0.56
Renal disease	15 (6.3%)	11 (7.6%)	4 (4.3%)	0.31
Metabolic disease	6 (2.5%)	4 (2.8%)	2 (2.2%)	0.77
Other	3 (1.3%)	2 (1.4%)	1 (1.1%)	0.84
Comorbidity, *n* (%)				
Hypertension	139 (58.4%)	92 (63.4%)	47 (50.5%)	**0.049**
Malignant disease	113 (47.5%)	65 (44.8%)	48 (51.6%)	0.31
Chronic kidney disease	81 (34.0%)	49 (33.8%)	32 (34.4%)	0.92
Coronary heart disease	54 (22.7%)	35 (24.1%)	19 (20.4%)	0.51
Diabetes	43 (18.1%)	28 (19.3%)	15 (16.1%)	0.53
Congestive heart failure	45 (18.9%)	31 (21.4%)	14 (15.1%)	0.22
COPD	28 (11.8%)	19 (13.1%)	9 (9.7%)	0.42
Peripheral arterial disease	26 (10.9%)	16 (11.0%)	10 (10.8%)	0.95
Stroke	27 (11.3%)	16 (11.0%)	11 (11.8%)	0.85
Dementia	11 (4.6%)	8 (5.5%)	3 (3.2%)	0.41

Groups based on high or low tryptophan plasma levels. We compared frequencies using Person’s chi-squared test and continuous variables using a two-sample t-test.COPD, Chronic obstructive pulmonary disease; NRS 2002, Nutritional Risk Screening 2002. The values in bold indicate that they are statistically significant (*p* value of <0.05).

### 3.2 Association of nutritional parameters and inflammation with tryptophan metabolites

In a first step, we investigated the association of nutritional parameters and inflammation with tryptophan metabolites (Supplementary Table 1). Higher body mass index was positively associated with tryptophan levels in a linear regression model (Coef 3.52 [95% CI 1.16–5.88, *p* 0.004]). Furthermore, disease severity was associated with low tryptophan plasma levels (<36.2 μmol/L) in a logistic regression model (OR 2.12 [95% CI 1.26–3.56, *p* 0.005]). No correlation was found with inflammation (assessed by CRP) or nutritional risk based on the NRS 2002 total score.

### 3.3 Association of tryptophan metabolites with mortality and clinical outcomes

**Figure 2:**
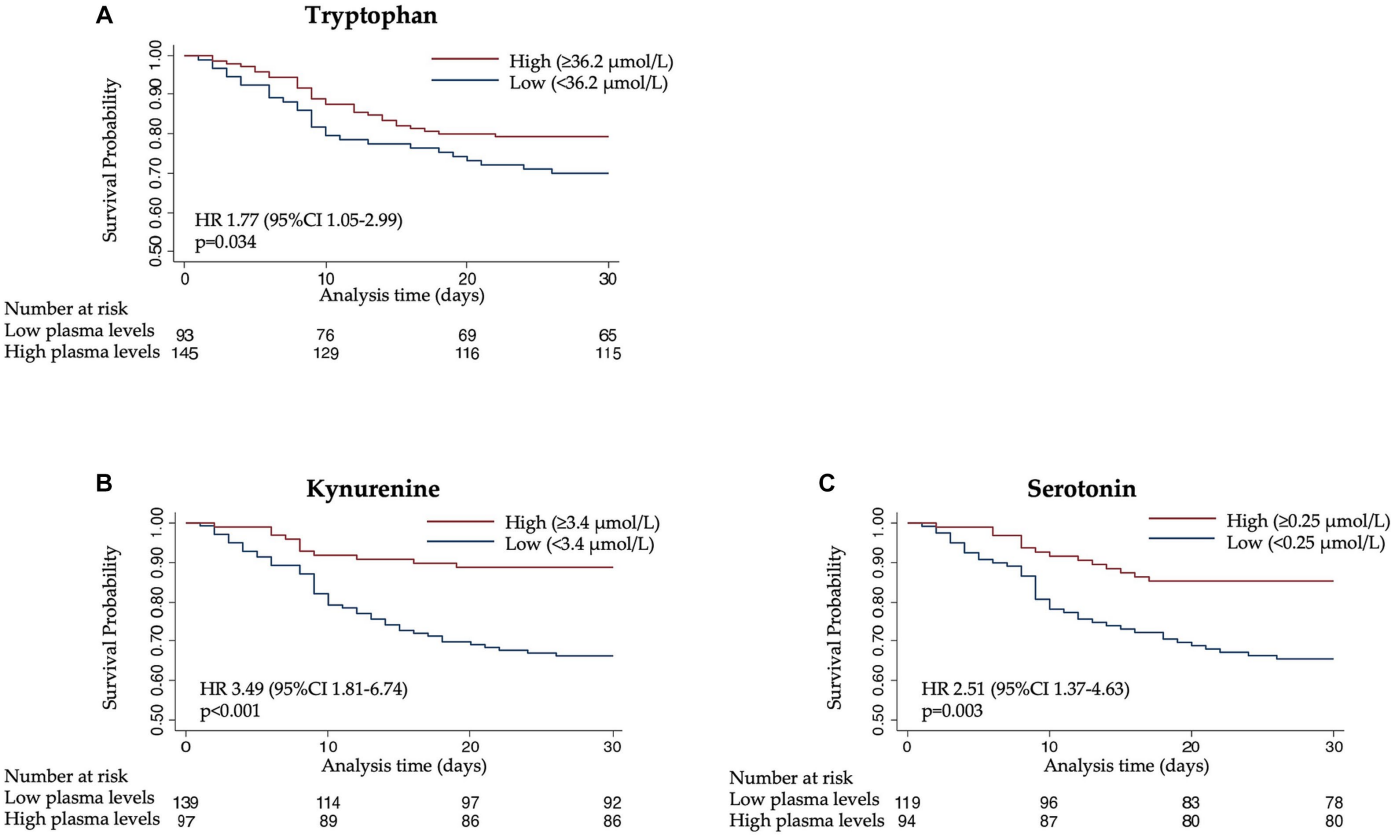
Kaplan–Meier estimate for time to death within 30 days according to **(A)** tryptophan, **(B)** kynurenine, and **(C)** serotonin levels.

Next, to understand prognostic implications, we investigated the association of tryptophan and its metabolites with clinical outcomes (Table 2). Low plasma levels of all metabolites were associated with an increased risk of 30-day mortality in a multivariate cox regression model with adjusted HRs of 1.77 [95% CI 1.05–2.99, *p* 0.034] for tryptophan, 3.49 [95% CI 1.81–6.74, *p* < 0.001] for kynurenine, and 2.51 [95% CI 1.37–4.63, *p* 0.003] for serotonin (Figure 2). Likewise, low values of the calculated enzymes TPH and IDO were associated with 30-day mortality in a univariate model with HRs of 2.41 [95% CI 1.39–4.17, *p* 0.002] for TPH and 1.73 [95% CI 1.03–2.90, *p* 0.038] for IDO. These results remained consistent in an adjusted model for TPH, but not for IDO.

**Table 2:** Prognostic value of tryptophan metabolites to predict clinical outcomes.

All-cause mortality	Low plasma levels	High plasma levels	HR^*^ (95% CI)	*p* value
30-day all-cause mortality				
Tryptophan	28/93 (30.1%)	30/145 (20.7%)	1.77 (1.05–2.99)	**0.034**
Kynurenine	47/139 (33.8%)	11/97 (11.3%)	3.49 (1.81–6.74)	**<0.001**
Serotonin	41/119 (34.5%)	14/94 (14.9%)	2.51 (1.37–4.63)	**0.003**
IDO	32/101 (31.7%)	26/135 (19.3%)	1.59 (0.94–2.69)	0.084
TPH	35/96 (36.5%)	20/117 (17.1%)	2.52 (1.45–4.38)	**0.001**
180-day all-cause mortality				
Tryptophan	48/93 (51.6%)	54/145 (37.2%)	1.74 (1.16–2.61)	**0.007**
Kynurenine	67/139 (48.2%)	34/97 (35.1%)	1.77 (1.17–2.69)	**0.007**
Serotonin	59/119 (49.6%)	33/94 (35.1%)	1.63 (1.06–2.51)	**0.026**
IDO	45/101 (44.6%)	56/135 (41.5%)	1.15 (0.77–1.71)	0.499
TPH	46/96 (47.9%)	46/117 (39.3%)	1.46 (0.97–2.20)	0.073
3-year all-cause mortality				
Tryptophan	60/93 (64.5%)	83/145 (57.2%)	1.41 (1.00–1.98)	**0.049**
Kynurenine	87/139 (62.6%)	55/97 (56.7%)	1.50 (1.06–2.12)	**0.021**
Serotonin	77/119 (64.7%)	52/94 (55.3%)	1.35 (0.95–1.93)	0.093
IDO	61/101 (60.4%)	81/135 (60.0%)	1.09 (0.77–1.53)	0.634
TPH	62/96 (64.6%)	67/117 (57.3%)	1.31 (0.93–1.86)	0.127
Other clinical outcomes	Low plasma levels	High plasma levels	OR^*^ (95% CI)	*p* value
Adverse events within 30-day				
Tryptophan	40/93 (43.0%)	53/145 (36.6%)	1.39 (0.97–1.00)	0.247
Kynurenine	68/139 (48.9%)	24/97 (24.7%)	3.15 (1.72–5.77)	**<0.001**
Serotonin	53/119 (44.5%)	32/94 (34.0%)	1.47 (0.82–2.65)	0.200
IDO	45/101 (44.6%)	47/135 (34.8%)	1.44 (0.82–2.53)	0.200
TPH	44/96 (45.8%)	41/117 (35.0%)	1.56 (0.87–2.79)	0.140
Major complications within 30-day				
Tryptophan	9/93 (9.7%)	13/145 (9.0%)	1.11 (0.45–2.72)	0.826
Kynurenine	16/139 (11.5%)	6/97 (6.2%)	1.99 (0.75–5.33)	0.169
Serotonin	12/119 (10.1%)	7/94 (7.5%)	1.32 (0.49–3.52)	0.585
IDO	10/101 (9.9%)	12/135 (8.9%)	1.12 (0.46–2.74)	0.805
TPH	11/96 (11.5%)	8/117 (6.8%)	1.66 (0.64–4.34)	0.300
Barthel decline >10%				
Tryptophan	29/93 (31.2%)	36/145 (24.8%)	1.58 (0.84–2.97)	0.157
Kynurenine	48/139 (34.5%)	17/97 (17.5%)	2.78 (1.40–5.53)	**0.003**
Serotonin	40/119 (33.6%)	20/94 (21.3%)	1.73 (0.88–3.39)	0.109
IDO	35/101 (34.7%)	30/135 (22.2%)	1.82 (0.97–3.43)	0.062
TPH	33/96 (34.4%)	27/117 (23.1%)	1.73 (0.90–3.33)	0.101

Groups based on high or low metabolite plasma levels. Data are number of events (%). *HR and *OR were adjusted for comorbidity, age, sex, randomization group and nutritional risk Screening 2002 total score.CI, Confidence interval; HR, Hazard ratio; IDO, Indoleamine 2,3-dioxygenase; OR, Odds ratio; and TPH, Tryptophan hydroxylase. The values in bold indicate that they are statistically significant (*p* value of <0.05).

Regarding the long-term outcome, low plasma levels of tryptophan, kynurenine, and serotonin were associated with increased mortality after 180 days with adjusted HRs of 1.74 [95% CI 1.16–2.61, *p* 0.007] for tryptophan, 1.77 [95% CI 1.17–2.69, *p* 0.007] for kynurenine, and 1.63 [95% CI 1.06–2.51, *p* 0.026] for serotonin.

Regarding the combined endpoint of adverse events, we found significant association with low values of kynurenine (adjusted OR 3.15 [95% CI 1.72–5.77, *p* < 0.001]) but not with other metabolites.

### 3.4 Association of tryptophan metabolites with response to nutritional support

**Figure 3:**
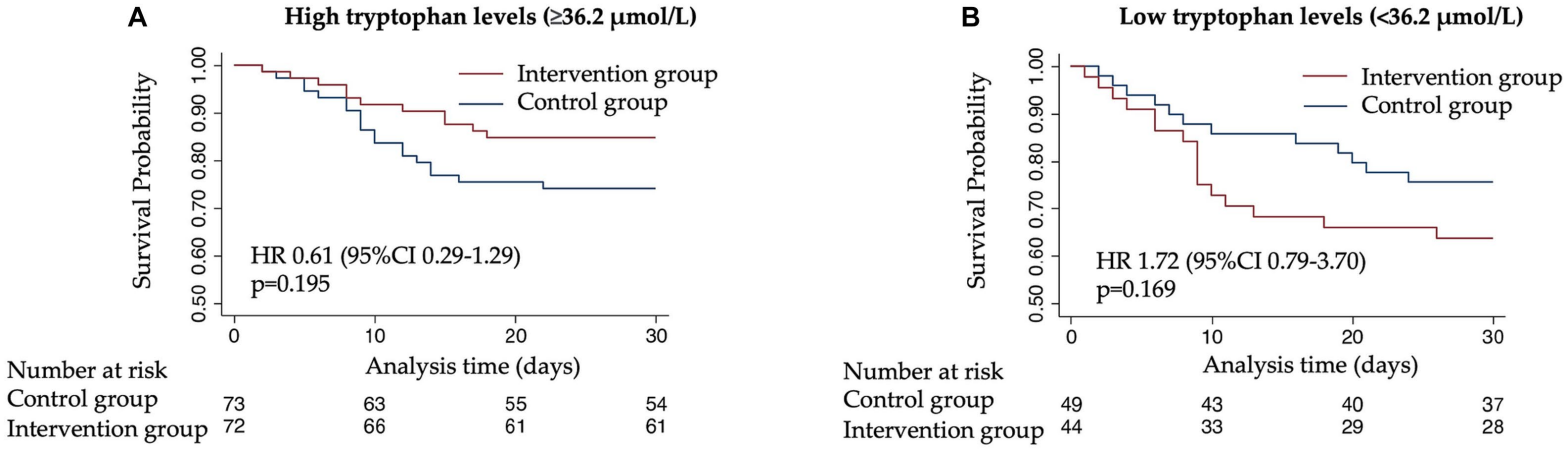
Kaplan–Meier estimate for time to death within 30 days according to randomization group. **(A)** 30-day mortality among patients with high tryptophan plasma levels (≥36.2 μmol/L). **(B)** 30-day mortality among patients with low tryptophan plasma levels (<36.2 μmol/L).

**Figure 4:**
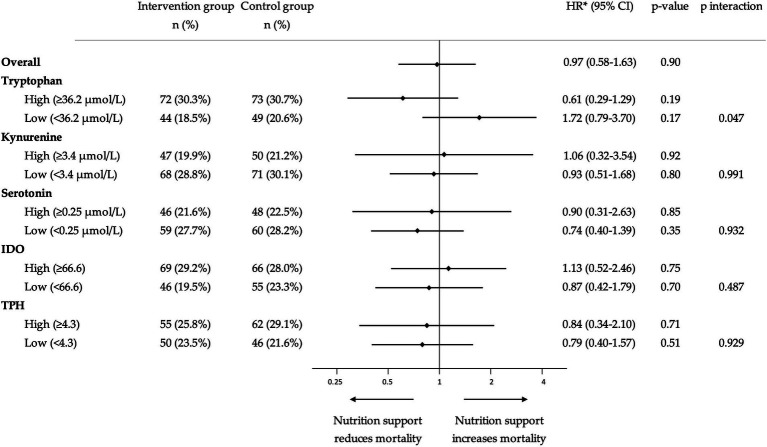
Hazard ratio plot for 30-day all-cause mortality, subgroup analysis for response to nutritional support. Data are presented in a logarithmic scale. ^*^HR were adjusted for comorbidity, age, sex, randomization group, and Nutritional Risk Screening 2002 total score. HR, Hazard ratio; IDO, Indoleamine 2,3-dioxygenase; and TPH, Tryptophan hydroxylase.

To understand whether the treatment response to nutritional support would differ according to admission tryptophan levels, we compared clinical outcomes based on the initial randomization (intervention vs. control) in patients stratified by high or low levels of tryptophan and its metabolites. The nutritional support intervention showed a trend toward a beneficial effect and lower risk of 30-day mortality in patients with high tryptophan levels (adjusted HR 0.61 [95% CI 0.29–1.29, *p* 0.195]), but not in patients with low tryptophan levels (adjusted HR 1.72 [95% CI 0.79–3.70, *p* 0.169]) (Figure 3). This difference in effect was significant in interaction analysis (*p* for interaction 0.047). Similar analyses with the other metabolites and enzymes under investigation (kynurenine, serotonin, IDO, and TPH) did not show similar results regarding the effectiveness of the response to nutritional therapy (Figure 4).

## 4 Discussion

In this secondary analysis of a one-center subset from a large, multicenter, randomized, controlled clinical trial, we investigated the prognostic implications of tryptophan pathway metabolites regarding clinical outcomes and response to nutritional support among patients at nutritional risk. This study has several key findings. First, the availability of tryptophan was in general reduced in our malnourished patient cohort. Second, low levels of tryptophan were associated with disease severity, but not with nutritional intake or nutritional risk based on the NRS 2002 total score. Third, low levels of tryptophan, kynurenine, and serotonin were also associated with an increased risk in 30-day and 180-day mortality, respectively. This suggest that these metabolites provide prognostic information in patients at nutritional risk that is independent from their nutritional status. Finally, although patients with low tryptophan levels had higher risk for mortality, nutritional support appeared to have the smallest effects in these patients as compared to patients with higher tryptophan levels.

Our data differ from previously conducted studies in pneumonia, COPD, and sepsis, where an activation of the kynurenine and serotonin pathways was associated with adverse outcomes (22, 24, 38). Metabolic pathways are complex and can be influenced by several clinical conditions. The selection of our rather heterogenous medical patient cohort at risk for malnutrition with various underlying diseases might explain these differences. Deficient protein intake leads to low tryptophan plasma levels and hence its availability. Although we could not demonstrate an association of tryptophan with the NRS 2002 total score, tryptophan levels were low in our patient cohort compared to healthy volunteers in France (median tryptophan levels 40.1 vs. 62.7 μmol/L) (15). Also, in comparison to a study with a COPD patient cohort with similar age distribution and comorbidities, tryptophan levels were lower in our malnourished patient cohort (40.1 vs. 50.3 μmol/L) (24). This supports the hypothesis of low tryptophan levels due to malnutrition, and thus its decreased availability for the subsequent metabolic pathways, likely leading to a general downregulation of the involved enzymes. Furthermore, tryptophan levels depend on age and decrease over time (15). This may have an influence on tryptophan levels in our rather older patient population. Several underlying conditions can also influence tryptophan levels and the activity of the enzymes. High IDO activity and thus activation of the kynurenine pathway was associated with inflammation and malignant disease in previous studies (21, 22). In our study, we found an association between infectious diseases and low tryptophan levels, probably due to an activation of the kynurenine pathway and IDO activity. However, no association was found with levels of inflammation (CRP). Due to the sample size, we did not further explore effects in subgroup analyses regarding the underlying diseases and outcomes.

An important and new finding of this study was that, although low tryptophan levels were associated with higher mortality, nutritional support in those patients with low tryptophan levels was less effective in improving outcomes compared to patients with higher levels. This finding is comparable with data observed in critically ill patients, where only little benefit from full-replacement feeding could be shown in several clinical trials (10, 11). In fact, high nutritional intake during severe illness has been suggested to reduce autophagy, a mechanism important for cell detoxication during illness, which may explain lack of effect in these patients (39). Similarly, in our patient cohort, low tryptophan levels may identify patients with severe disease and high metabolic stress in whom reaching protein and caloric goals may not translate to clinical benefit. Whether low tryptophan levels are a consequence or cause of the observed poor outcomes and thus specific nutritional intervention including tryptophan could have beneficial effects in this highly vulnerable patient population, needs to be further investigated. To the best of our knowledge, there are no treatment studies answering the question of whether tryptophan supplementation or modification of the tryptophan/kynurenine pathway have an impact on survival.

This secondary analysis has several strengths and limitations. The main strength of this study includes the well characterized patient cohort, the randomized design, and the prospectively collected short- and long-term outcomes. Thus, we were able to adjust our analysis for potential confounders including comorbidities and nutritional parameters. We had consistent results also after adjusting for those confounders. To our knowledge, this is the first analysis based on a randomized, controlled clinical trial to examine the prognostic value of tryptophan metabolites and their role as predictors for the effectiveness of nutritional support. However, we are aware of the limitations in our analysis. Metabolite measurements were only available in a small subset of the original patient cohort, and blood samples were collected from one study center only. This limits the power and external validity of the analysis. In light of the limited sample size and the exploratory nature of this study, we also did not adjust for multiple testing. Given the exploratory nature of this secondary analysis, this study should be considered hypothesis generating rather than definitive. There are also limitations regarding the used metabolomic kit. So far, it has been mainly used for research purposes and lacks well validated reference values. Due to internal validation, results can differ between laboratories and comparison of the measured values are only possible to a limited extend. Furthermore, the enzymes IDO and TPH could not be measured and were therefore estimated through the ratio of kynurenine/ serotonin to tryptophan, making them vulnerable to errors. Since plasma levels of tryptophan metabolites were only measured upon study inclusion, no statement could be made regarding the influence of nutritional therapy on the metabolites.

In conclusion, our data suggest that levels of tryptophan, kynurenine, and serotonin are low in patients at nutritional risk, and strongly associated with mortality and adverse outcomes. However, nutritional support for patients with tryptophan levels below a calculated cut point was less effective in improving these outcomes. This may be due to an association of disease severity in patients with low tryptophan levels. Whether specific nutritional interventions including tryptophan may have beneficial effects in this highly vulnerable patient population needs to be analyzed. Further prospective studies are required to validate our results, evaluate the impact of nutritional support on levels of tryptophan metabolites, and whether therapeutic modulations of these pathways have positive effects on outcomes.

## Data availability statement

The data analyzed in this study are subject to the following licenses/restrictions: Data described in the manuscript, codebook, and analytic code are available upon request after other secondary projects related to the study are completed. Requests to access these datasets should be directed to schuetzph@gmail.com.

## Ethics statement

The studies involving humans were approved by Ethics Committee of Northwest and Central Switzerland (EKNZ). The studies were conducted in accordance with the local legislation and institutional requirements. The participants provided their written informed consent to participate in this study.

## Author contributions

JR: Formal analysis, Visualization, Writing – original draft, Writing – review & editing. CW: Conceptualization, Formal analysis, Investigation, Writing – review & editing. FS: Formal analysis, Writing – review & editing. RL: Formal analysis, Writing – review & editing. PT: Investigation, Writing – review & editing. PN: Investigation, Writing – review & editing. LB: Investigation, Writing – review & editing. ZS: Funding acquisition, Investigation, Writing – review & editing. BM: Funding acquisition, Investigation, Writing – review & editing. PS: Conceptualization, Funding acquisition, Investigation, Project administration, Writing – review & editing.

## References

[1] Felder S Lechtenboehmer C Bally M Fehr R Deiss M Faessler L. Association of nutritional risk and adverse medical outcomes across different medical inpatient populations. Nutrition. (2015) 31:1385–93. doi: 10.1016/j.nut.2015.06.007, PMID: 26429660

[2] Imoberdorf R Meier R Krebs P Hangartner PJ Hess B Stäubli M. Prevalence of undernutrition on admission to Swiss hospitals. Clin Nutr. (2010) 29:38–41. doi: 10.1016/j.clnu.2009.06.005, PMID: 19573958

[3] Schuetz P Seres D Lobo DN Gomes F Kaegi-Braun N Stanga Z. Management of disease-related malnutrition for patients being treated in hospital. Lancet. (2021) 398:1927–38. doi: 10.1016/S0140-6736(21)01451-3, PMID: 34656286

[4] Deutz NE Matheson EM Matarese LE Luo M Baggs GE Nelson JL. Readmission and mortality in malnourished, older, hospitalized adults treated with a specialized oral nutritional supplement: a randomized clinical trial. Clin Nutr. (2016) 35:18–26. doi: 10.1016/j.clnu.2015.12.010, PMID: 26797412

[5] Bonilla-Palomas JL Gamez-Lopez AL Castillo-Dominguez JC Moreno-Conde M Lopez Ibanez MC Alhambra Exposito R. Nutritional intervention in malnourished hospitalized patients with Heart failure. Arch Med Res. (2016) 47:535–40. doi: 10.1016/j.arcmed.2016.11.005 28262195

[6] Schuetz P Fehr R Baechli V Geiser M Deiss M Gomes F. Individualised nutritional support in medical inpatients at nutritional risk: a randomised clinical trial. Lancet. (2019) 393:2312–21. doi: 10.1016/S0140-6736(18)32776-4 31030981

[7] Bargetzi A Emmenegger N Wildisen S Nickler M Bargetzi L Hersberger L. Admission kidney function is a strong predictor for the response to nutritional support in patients at nutritional risk. Clin Nutr. (2021) 40:2762–71. doi: 10.1016/j.clnu.2021.03.013, PMID: 33933742

[8] Merker M Felder M Gueissaz L Bolliger R Tribolet P Kagi-Braun N. Association of Baseline Inflammation with Effectiveness of nutritional support among patients with disease-related malnutrition: a secondary analysis of a randomized clinical trial. JAMA Netw Open. (2020) 3:e200663. doi: 10.1001/jamanetworkopen.2020.0663, PMID: 32154887 PMC7064875

[9] Bretscher C Buergin M Gurzeler G Kagi-Braun N Gressies C Tribolet P. Association between prealbumin, all-cause mortality, and response to nutrition treatment in patients at nutrition risk: secondary analysis of a randomized controlled trial. JPEN J Parenter Enteral Nutr. (2023) 47:408–19. doi: 10.1002/jpen.2470, PMID: 36587281

[10] Vanderheyden S Casaer MP Kesteloot K Simoens S De Rijdt T Peers G. Early versus late parenteral nutrition in ICU patients: cost analysis of the EPaNIC trial. Crit Care. (2012) 16:R96. doi: 10.1186/cc11361, PMID: 22632574 PMC3580642

[11] National Heart L Blood Institute Acute Respiratory Distress Syndrome Clinical Trials, N Rice TW Wheeler AP Thompson BT Steingrub J. Initial trophic vs full enteral feeding in patients with acute lung injury: the EDEN randomized trial. JAMA. (2012) 307:795–803. doi: 10.1001/jama.2012.137, PMID: 22307571 PMC3743415

[12] Badawy AA. Kynurenine pathway of tryptophan metabolism: regulatory and functional aspects. Int J Tryptophan Res. (2017) 10:1178646917691938. doi: 10.1177/1178646917691938, PMID: 28469468 PMC5398323

[13] Badawy AA. Tryptophan availability for kynurenine pathway metabolism across the life span: control mechanisms and focus on aging, exercise, diet and nutritional supplements. Neuropharmacology. (2017) 112:248–63. doi: 10.1016/j.neuropharm.2015.11.015, PMID: 26617070

[14] Truswell AS Hansen JD Wannenburg P. Plasma tryptophan and other amino acids in pellagra. Am J Clin Nutr. (1968) 21:1314–20. doi: 10.1093/ajcn/21.11.1314, PMID: 4235335

[15] Trabado S Al-Salameh A Croixmarie V Masson P Corruble E Feve B. The human plasma-metabolome: reference values in 800 French healthy volunteers; impact of cholesterol, gender and age. PLoS One. (2017) 12:e0173615. doi: 10.1371/journal.pone.0173615, PMID: 28278231 PMC5344496

[16] Bender DA. Biochemistry of tryptophan in health and disease. Mol Asp Med. (1983) 6:101–97. doi: 10.1016/0098-2997(83)90005-5, PMID: 6371429

[17] Le Floc'h N Otten W Merlot E. Tryptophan metabolism, from nutrition to potential therapeutic applications. Amino Acids. (2011) 41:1195–205. doi: 10.1007/s00726-010-0752-7, PMID: 20872026

[18] Aune TM Pogue SL. Inhibition of tumor cell growth by interferon-gamma is mediated by two distinct mechanisms dependent upon oxygen tension: induction of tryptophan degradation and depletion of intracellular nicotinamide adenine dinucleotide. J Clin Invest. (1989) 84:863–75. doi: 10.1172/jci114247, PMID: 2503544 PMC329730

[19] Pfefferkorn ER. Interferon gamma blocks the growth of toxoplasma gondii in human fibroblasts by inducing the host cells to degrade tryptophan. Proc Natl Acad Sci USA. (1984) 81:908–12. doi: 10.1073/pnas.81.3.908, PMID: 6422465 PMC344948

[20] Mellor AL Keskin DB Johnson T Chandler P Munn DH. Cells expressing indoleamine 2,3-dioxygenase inhibit T cell responses. J Immunol. (2002) 168:3771–6. doi: 10.4049/jimmunol.168.8.3771 11937528

[21] Munn DH Mellor AL. IDO and tolerance to tumors. Trends Mol Med. (2004) 10:15–8. doi: 10.1016/j.molmed.2003.11.003 14720581

[22] Meier MA Ottiger M Vogeli A Steuer C Bernasconi L Thomann R. Activation of the tryptophan/serotonin pathway is associated with severity and predicts outcomes in pneumonia: results of a long-term cohort study. Clin Chem Lab Med. (2017) 55:1060–9. doi: 10.1515/cclm-2016-0912, PMID: 28076309

[23] Suzuki Y Suda T Furuhashi K Suzuki M Fujie M Hahimoto D. Increased serum kynurenine/tryptophan ratio correlates with disease progression in lung cancer. Lung Cancer. (2010) 67:361–5. doi: 10.1016/j.lungcan.2009.05.001, PMID: 19487045

[24] Meier MA Ottiger M Vogeli A Steuer C Bernasconi L Thomann R. Activation of the serotonin pathway is associated with poor outcome in COPD exacerbation: results of a long-term cohort study. Lung. (2017) 195:303–11. doi: 10.1007/s00408-017-0004-7, PMID: 28434116

[25] Mohammad-Zadeh LF Moses L Gwaltney-Brant SM. Serotonin: a review. J Vet Pharmacol Ther. (2008) 31:187–99. doi: 10.1111/j.1365-2885.2008.00944.x 18471139

[26] Vikenes K Farstad M Nordrehaug JE. Serotonin is associated with coronary artery disease and cardiac events. Circulation. (1999) 100:483–9. doi: 10.1161/01.cir.100.5.483 10430761

[27] Tanaka T Mori M Sekino M Higashijima U Takaki M Yamashita Y. Impact of plasma 5-hydroxyindoleacetic acid, a serotonin metabolite, on clinical outcome in septic shock, and its effect on vascular permeability. Sci Rep. (2021) 11:14146. doi: 10.1038/s41598-021-93649-z, PMID: 34238999 PMC8266895

[28] Kondrup J Rasmussen HH Hamberg OLE Stanga Z. Nutritional risk screening (NRS 2002): a new method based on an analysis of controlled clinical trials. Clin Nutr. (2002) 22:321–36. doi: 10.1016/s0261-5614(02)00214-5, PMID: 12765673

[29] Bounoure L Gomes F Stanga Z Keller U Meier R Ballmer P. Detection and treatment of medical inpatients with or at-risk of malnutrition: suggested procedures based on validated guidelines. Nutrition. (2016) 32:790–8. doi: 10.1016/j.nut.2016.01.019 27160498

[30] Gomes F Schuetz P Bounoure L Austin P Ballesteros-Pomar M Cederholm T. ESPEN guidelines on nutritional support for polymorbid internal medicine patients. Clin Nutr. (2018) 37:336–53. doi: 10.1016/j.clnu.2017.06.025, PMID: 28802519

[31] Illig T Gieger C Zhai G Romisch-Margl W Wang-Sattler R Prehn C. A genome-wide perspective of genetic variation in human metabolism. Nat Genet. (2010) 42:137–41. doi: 10.1038/ng.507, PMID: 20037589 PMC3773904

[32] Weinberger KM. Metabolomics in diagnosing metabolic diseases. Ther Umsch. (2008) 65:487–91. doi: 10.1024/0040-5930.65.9.487, PMID: 18791962

[33] Yet I Menni C Shin SY Mangino M Soranzo N Adamski J. Genetic influences on metabolite levels: a comparison across Metabolomic platforms. PLoS One. (2016) 11:e0153672. doi: 10.1371/journal.pone.0153672, PMID: 27073872 PMC4830611

[34] Siskos AP Jain P Römisch-Margl W Bennett M Achaintre D Asad Y. Interlaboratory reproducibility of a targeted metabolomics platform for analysis of human serum and plasma. Anal Chem. (2017) 89:656–65. doi: 10.1021/acs.analchem.6b02930, PMID: 27959516 PMC6317696

[35] Widner B Werner ER Schennach H Wachter H Fuchs D. Simultaneous measurement of serum tryptophan and kynurenine by HPLC. Clin Chem. (1997) 43:2424–6. doi: 10.1093/clinchem/43.12.2424, PMID: 9439467

[36] Liu X. Classification accuracy and cut point selection. Stat Med. (2012) 31:2676–86. doi: 10.1002/sim.4509 22307964

[37] Charlson ME Pompei P Ales KL MacKenzie CR. A new method of classifying prognostic comorbidity in longitudinal studies: development and validation. J Chronic Dis. (1987) 40:373–83. doi: 10.1016/0021-9681(87)90171-8, PMID: 3558716

[38] Rogers AJ McGeachie M Baron RM Gazourian L Haspel JA Nakahira K. Metabolomic derangements are associated with mortality in critically ill adult patients. PLoS One. (2014) 9:e87538. doi: 10.1371/journal.pone.0087538, PMID: 24498130 PMC3907548

[39] Gunst J Derese I Aertgeerts A Ververs EJ Wauters A Van den Berghe G. Insufficient autophagy contributes to mitochondrial dysfunction, organ failure, and adverse outcome in an animal model of critical illness. Crit Care Med. (2013) 41:182–94. doi: 10.1097/CCM.0b013e3182676657, PMID: 23222264

