# Supplementary material for: Association of tryptophan pathway metabolites with mortality and effectiveness of nutritional support among patients at nutritional risk: secondary analysis of a randomized clinical trial
Source: Front Nutr. 2024 Feb 15;11:1335242. doi: 10.3389/fnut.2024.1335242 (PMC10902466; doi:10.3389/fnut.2024.1335242)
Supplement: Supplementary file 1 [file Table_1.DOCX]

**Supplemental table 1.** Association of nutritional parameters and inflammation with tryptophan metabolites

|  |  | **OR (95% CI)  (outcome = low metabolite levels (< cut point))** | **p-value** | **Coeff (95% CI)  (outcome = continuous metabolite levels)** | **p-value** |
| --- | --- | --- | --- | --- | --- |
| **Nutritional parameters** (categorical predictor variables) | | | |  |  |
| NRS 2002 total score | |  |  |  |  |
|  | Tryptophan | 1.08 (0.78 - 1.49) | 0.653 | -0.96 (-3.59 - 1.67) | 0.472 |
|  | Kynurenine | 1.26 (0.91 - 1.74) | 0.168 | -0.27 (-0.64 - 0.09) | 0.140 |
|  | Serotonin | 0.95 (0.67 - 1.33) | 0.759 | 0.08 (-0.03 - 0.19) | 0.140 |
|  | IDO | 0.95 (0.69 - 1.31) | 0.746 | -2.21 (-18.95 - 14.53) | 0.795 |
|  | TPH | 0.93 (0.66 - 1.30) | 0.661 | 1.93 (-0.69 - 4.55) | 0.148 |
| Nutritional intake | |  |  |  |  |
|  | Tryptophan | 1.35 (1.00 - 1.82) | 0.047 | 0.81 (-1.56 - 3.18) | 0.499 |
|  | Kynurenine | 1.12 (0.84 - 1.50) | 0.445 | 0.05 (-0.28 - 0.39) | 0.745 |
|  | Serotonin | 0.86 (0.63 - 1.17) | 0.329 | -0.11 (-0.21 - -0.01) | 0.028 |
|  | IDO | 1.02 (0.76 - 1.36) | 0.911 | -0.13 (-15.28 - 15.01) | 0.986 |
|  | TPH | 0.78 (0.57 - 1.06) | 0.113 | -2.90 (-5.23 - -0.58) | 0.014 |
| Body mass index | |  |  |  |  |
|  | Tryptophan | 0.77 (0.57 - 1.04) | 0.093 | 3.52 (1.16 - 5.88) | **0.004** |
|  | Kynurenine | 0.86 (0.64 - 1.16) | 0.325 | 0.23 (-0.10 - 0.56) | 0.169 |
|  | Serotonin | 0.98 (0.72 - 1.33) | 0.904 | -0.11 (-0.21 - -0.02) | 0.022 |
|  | IDO | 0.87 (0.65 - 1.16) | 0.343 | -7.10 (-22.33 - 8.12) | 0.359 |
|  | TPH | 1.09 (0.81 - 1.48) | 0.560 | -3.41 (-5.70 - -1.11) | **0.004** |
| Weight loss | |  |  |  |  |
|  | Tryptophan | 0.97 (0.75 - 1.24) | 0.790 | 0.49 (-1.53 - 2.51) | 0.632 |
|  | Kynurenine | 1.19 (0.93 - 1.54) | 0.170 | -0.25 (-0.53 - 0.03) | 0.083 |
|  | Serotonin | 1.02 (0.79 - 1.32) | 0.868 | -0.02 (-0.10 - 0.07) | 0.710 |
|  | IDO | 1.04 (0.82 - 1.33) | 0.738 | -3.21 (-16.30 - 9.87) | 0.629 |
|  | TPH | 0.95 (0.73 - 1.22) | 0.669 | -0.44 (-2.46 - 1.58) | 0.669 |
| Disease severity | |  |  |  |  |
|  | Tryptophan | 2.12 (1.26 - 3.56) | **0.005** | -3.56 (-7.68 - 0.56) | 0.090 |
|  | Kynurenine | 1.65 (0.98 - 2.79) | 0.060 | -0.13 (-0.70 - 0.45) | 0.660 |
|  | Serotonin | 1.44 (0.84 - 2.47) | 0.185 | -0.15 (-0.32 - 0.02) | 0.093 |
|  | IDO | 0.96 (0.58 - 1.59) | 0.864 | 15.49 (-10.80 - 41.78) | 0.247 |
|  | TPH | 1.03 (0.61 - 1.76) | 0.903 | -3.28 (-7.36 - 0.80) | 0.115 |
| **Inflammation** (binary predictor variable) | | | |  |  |
| CRP | |  |  |  |  |
|  | Tryptophan | 0.91 (0.53-1.55) | 0.715 | 1.39 (-2.93 - 5.71) | 0.528 |
|  | Kynurenine | 1.20 (0.70-2.04) | 0.514 | 0.13 (-0.47-0.74) | 0.663 |
|  | Serotonin | 1.15 (0.66-2.01) | 0.620 | -0.09 (-0.27 - 0.09) | 0.337 |
|  | IDO | 1.25 (0.74-2.12) | 0.409 | 9.81 (-17.75-37.37) | 0.484 |
|  | TPH | 0.89 (0.51-1.55) | 0.669 | -1.12 (-5.41-3.18) | 0.609 |

Odds ratios were calculated with a logistic regression. Coefficients were calculated with a linear regression. The nutritional parameters are categorical variables. CRP was defined as binary variable (cut point </ ≥ 100mg/l). OR >1 indicates an association of higher NRS 2002 total score, nutritional intake, body mass index, weight loss, disease severity or CRP level with low metabolite plasma levels. Coeff >0 indicates a positive correlation of the scores of the predictor variables with the continuous metabolite plasma levels. CI = confidence interval. CRP = C-reactive protein. Coef = coefficient. IDO = indoleamine 2,3-dioxygenase. NRS 2002 = Nutritional Risk Screening 2002. OR = odds ratio. TPH = tryptophan hydroxylase.
